# Postbiotics of *Lacticaseibacillus paracasei* CECT 9610 and *Lactiplantibacillus plantarum* CECT 9608 attenuates store‐operated calcium entry and FAK phosphorylation in colorectal cancer cells

**DOI:** 10.1002/1878-0261.13629

**Published:** 2024-03-21

**Authors:** Alvaro Macias‐Diaz, Jose J. Lopez, Maria Bravo, Isaac Jardín, Waldo Luis Garcia‐Jimenez, Francisco J. Blanco‐Blanco, Rosario Cerrato, Juan A. Rosado

**Affiliations:** ^1^ Department of Physiology (Cellular Physiology Research Group), Institute of Molecular Pathology Biomarkers (IMPB) Universidad de Extremadura Cáceres Spain; ^2^ Innovación en Gestión y Conservación de Ungulados S.L Cáceres Spain

**Keywords:** colorectal cancer, *Lacticaseibacillus paracasei*, *Lactiplantibacillus plantarum*, Orai1, STIM1, store‐operated Ca^2+^ entry

## Abstract

Store‐operated Ca^2+^ entry (SOCE) is a major mechanism for Ca^2+^ influx in colorectal cancer (CRC) cells. This mechanism, regulated by the filling state of the intracellular Ca^2+^ stores, is mediated by the endoplasmic reticulum Ca^2+^ sensors of the stromal interaction molecules (STIM) family [stromal interaction molecule 1 (STIM1) and STIM2] and the Ca^2+^‐release‐activated Ca^2+^ channels constituted by Orai family members, with predominance of calcium release‐activated calcium channel protein 1 (Orai1). CRC cells exhibit enhanced SOCE due to remodeling of the expression of the key SOCE molecular components. The enhanced SOCE supports a variety of cancer hallmarks. Here, we show that treatment of the colorectal adenocarcinoma cell lines HT‐29 and Caco‐2 with inanimate *Lacticaseibacillus paracasei* (CECT9610) and *Lactiplantibacillus plantarum* (CECT9608) attenuates SOCE, although no detectable effect is seen on SOCE in normal colon mucosa cells. The effect of *Lacticaseibacillus paracasei* and *Lactiplantibacillus plantarum* postbiotics was mediated by downregulation of Orai1 and STIM1, while the expression levels of Orai3 and STIM2 remained unaltered. Treatment of HT‐29 and Caco‐2 cells with inanimate *Lacticaseibacillus paracasei* and *Lactiplantibacillus plantarum* impairs *in vitro* migration by a mechanism likely involving attenuation of focal adhesion kinase (FAK) tyrosine phosphorylation. Cell treatment with the Orai1 inhibitor synta‐66 attenuates SOCE and prevents any further effect of *Lacticaseibacillus paracasei* and *Lactiplantibacillus plantarum* postbiotics. Together, our results indicate for the first time that *Lacticaseibacillus paracasei* and *Lactiplantibacillus plantarum* postbiotics selectively exert negative effects on Ca^2+^ influx through SOCE in colorectal adenocarcinoma cell lines, providing evidence for an attractive strategy against CRC.

## Graphical abstract

Colorectal cancer (CRC) cells exhibit altered store‐operated Ca^2+^ entry (SOCE), showing enhanced Ca^2+^ entry and expression of Orai1 and STIM1 proteins, which also increases cell migration. Exposure of CRC cells to *Lacticaseibacillus paracasei* and *Lactiplantibacillus plantarum* postbiotics reduces the expression of these proteins, resulting in a decrease of SOCE and, consequently, cell migration.

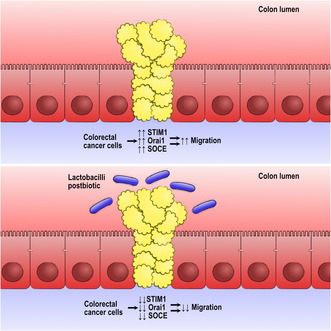

**Abbreviations**
[Ca^2+^]_c_
: cytosolic free‐Ca^2+^ concentration
AUC: area under the curve
Bax: Bcl‐2‐associated X protein
BSA: bovine serum albumin
CFU: colony forming units
CRC: colorectal cancer
DMEM: high‐glucose Dulbecco's modified Eagle's medium
EDTA: ethylenedinitrilotetraacetic acid
EGTA: ethylene glycol‐bis(2‐aminoethylether)‐*N*,*N*,*N*′,*N*′‐tetraacetic acid
FAK: focal adhesion kinase
fura‐2/AM: fura‐2 acetoxymethyl ester
HBS: HEPES‐buffered saline
HEPES: (4‐(2‐hydroxyethyl)piperazine‐1‐ethanesulfonic acid)
MMP‐9: matrix metalloproteinase‐9
SOCE: store‐operated Ca^2+^ entry
STIM: stromal interaction molecules
TBST: tris‐buffered saline with Tween
TG: thapsigargin
ZO‐1: zona occludens‐1

## 1 Introduction

Colorectal cancer (CRC) is among the most common malignancies and one of the deadliest cancer types. The development of CRC is associated to genetic features, environmental factors, including the composition of intestinal microbiota, and lifestyle [1]. CRC treatment includes laparoscopic surgery for focal localizations or resection in the case of metastatic disease, radiotherapy and chemotherapy based on the use of 5‐fluorouracil, platinum derivatives and cytostatic agents such as capecitabine, among others, alone or in combination (cancer.org, accessed on 22 August 2023). It has recently been reported that probiotics might help prevent CRC [2]. Based on the significant impact of gut microbiota in organs and systems physiology, supplements such as prebiotics, probiotics, and postbiotics have been reported to confer beneficial effects to health. Prebiotics are defined as substrates selectively used by host microorganisms that provide a health benefit [3]. Meanwhile, probiotics are live microorganisms that, in adequate amounts, confer health benefits [4] and the recently identified postbiotics include preparations of inanimate microorganisms and/or their components that confer health benefits to the host [5]. The term postbiotic includes a variety of metabolites including vitamins, amino acids, short‐chain fatty acids, microbial cell fragments, extracellular polysaccharides and organic acids, such as teichoic acid [6]. Postbiotics might act either by direct interaction with the host cells or indirectly modifying the host cells environment, i.e. inducing acidification, facilitating iron absorption [7] or reducing oxidative stress *in vivo* [8]. Cell‐free supernatants from *Lacticaseibacillus casei* and *Lacticaseibacillus rhamnosus GG* have been reported to reduce CRC cell invasion *in vitro* by reducing matrix metalloproteinase‐9 (MMP‐9) activity and expression of the tight junction protein zona occludens‐1 (ZO‐1) [9]. While the specific inhibitor has not been identified, it has been reported to be a macromolecule such as a protein, nucleic acid, or a polysaccharide [9]. β‐Glucans, a group of polymers of d‐glucose produced and released by bacteria forming a heterogeneous group of substances called exopolysaccharides, which are present in postbiotics, have been shown to interact with the leukocyte surface receptor Dectin‐1 leading to their activation and enhancing the immune response against cancer cells [10]. Enhanced anti‐cancer immunity has also been associated to bacterial lipoteichoic acid, which is present in the cell walls of Gram‐positive bacteria and released into the medium [11].

Colorectal cancer cells have been shown to exhibit different abnormalities in cytosolic Ca^2+^ homeostasis, including decreased accumulation of Ca^2+^ into the intracellular stores [12], probably associated to an early deficient expression of SERCA3 [13], abnormal Ca^2+^ uptake via the Na^+^/Ca^2+^‐exchanger, and enhanced store‐operated Ca^2+^ entry (SOCE) [14] a major mechanism for Ca^2+^ influx in CRC cells. SOCE is a ubiquitous mechanism for Ca^2+^ entry regulated by the filling state of the intracellular Ca^2+^ stores that mediates a vast variety of physiological process ranging from platelet aggregation [15, 16] or muscle contraction [17] to lactation [18] or immune responses [19, 20]. Thus, depletion of the intracellular Ca^2+^ stores, mainly the endoplasmic reticulum (ER), by physiological agonists results in a conformational change of the ER Ca^2+^ sensors STIM1 and STIM2 [21], single spanning transmembrane proteins, leading to the extension of the cytosolic region, which, in turn, facilitates the interaction with and activation of Orai channels in the plasma membrane [22, 23, 24, 25]. Orai1, and its mammalian homologs, Orai2 and Orai3, constitute the plasma membrane highly Ca^2+^ selective CRAC (Ca^2+^‐release‐activated Ca^2+^) channels, a hexameric structure where Orai1 plays a predominant role [26, 27]. In addition to the CRAC channels, STIM1 also modulates the activity of a less Ca^2+^ selective store‐operated channel involving, some TRPC proteins, predominantly TRPC1, called SOC (store‐operated Ca^2+^) channels [16, 28, 29].

Enhanced SOCE in CRC cells has been attributed to several abnormalities in the expression of the SOCE key molecular components. Among them, the expression of Orai proteins, as well as STIM1 and TRPC1 is enhanced at the transcript and protein level in the colorectal adenocarcinoma cell line HT‐29. By contrast, Sobradillo and coworkers reported that the expression of STIM2 is enhanced at the transcript level but reduced at the protein level [14]. Consistent with most of these observations, Orai proteins and both STIM isoforms, as well as TRPC1, 3, 4, and 6 are overexpressed at the transcript level in the colon carcinoma cell line HCT116 [30]. The results observed in culture cells are consistent with those reported in cells derived from primary tumors and metastatic lesions, where STIM1 and Orai3 are upregulated [31]. Among the mechanisms involved in the abnormal expression of SOCE components and enhanced SOCE, polyamines play a relevant role as treatment with α‐difluoromethylornithine, a suicide inhibitor of ornithine decarboxylase that limits polyamine synthesis, reverses the abnormal expression of SOCE molecular components [32]. Here we show for the first time that treatment of colorectal adenocarcinoma cells with postbiotics based on heat‐killed *Lacticaseibacillus paracasei* and *Lactiplantibacillus plantarum* attenuates SOCE via the modulation of Orai1 and STIM1 expression and inhibits the ability of these cells to migrate by impairing FAK tyrosine phosphorylation. These findings provide evidence for a potential effect of these postbiotics in anti‐colorectal cancer therapy.

## 2 Materials and methods

### 2.1 Reagents

Fura‐2 acetoxymethyl ester (fura‐2/AM) was from Molecular Probes (Leiden, the Netherlands). High‐glucose Dulbecco's modified Eagle's medium (DMEM), fetal bovine serum, trypsin, penicillin/streptomycin, SuperSignal® West Dura extended duration substrate reagent, Pierce™ BCA protein assay kit was purchased from ThermoFisher Scientific (Waltham, MA, USA). DharmaFECT kb transfection reagent was obtained from Horizon Discovery (Waterbeach, UK). Mouse monoclonal Anti‐GOK/Stim1 antibody (Clone 44/GOK; catalog number 610954, epitope: amino acids: 25–139 of human STIM1) was purchased from BD Biosciences (San Jose, CA, USA). Thapsigargin (TG), histamine, protein A agarose beads, HEPES (4‐(2‐hydroxyethyl)piperazine‐1‐ethanesulfonic acid), EGTA (ethylene glycol‐bis(2‐aminoethylether)‐*N*,*N*,*N*′,*N*′‐tetraacetic acid), synta66, EDTA (ethylenedinitrilotetraacetic acid), bovine serum albumin (BSA), sodium azide, sodium ascorbate, rabbit polyclonal anti‐Orai1 antibody (catalog number O8264, epitope: amino acids 288–301 of human Orai1), mouse monoclonal anti‐Orai3 antibody (Clone 1B4F1, epitope: 19 amino acids of the C‐Terminal domain), rabbit polyclonal anti‐β‐actin antibody (catalog number A2066, epitope: amino acids 365–375 of human β‐actin) and mouse monoclonal anti‐phosphotyrosine antibody (clone 4G10; catalog number 05‐321) were obtained from Sigma (St Louis, MO, USA). Rabbit polyclonal anti‐Orai2 antibody (catalog number TA306419, epitope: sequence localized in the C‐terminal region) was from Origiene (Herford, Germany). Rabbit polyclonal anti‐STIM2 antibody (catalog number 4917S) was from Cell Signaling Technology (Leiden, the Netherlands). Rabbit monoclonal anti‐FAK antibody (catalog number ab40794, epitope: within amino acids 700–800 of human FAK) was from Abcam (Cambridge, UK). Horseradish peroxidase‐conjugated goat anti‐mouse immunoglobulin G (IgG) antibody was from Jackson laboratories (West Grove, PA, USA). Fluorescent goat anti‐rabbit IgG StarBright Blue 700 antibody was from Bio‐Rad (Madrid, Spain). Complete EDTA‐free protease inhibitor cocktail tablets were from Roche Diagnostics GmbH (Mannheim, Germany). All other reagents were of an analytical grade as described previously [33].

### 2.2 Cell culture

Non‐tumoral NCM460 and colorectal adenocarcinoma HT‐29 cells were kindly provided by Carlos Villalobos (Institute of Molecular Biology and Genetics (IBGM), Valladolid, Spain) and Caco‐2 colorectal adenocarcinoma cells were provided by Mario Estevez (University of Extremadura, Cáceres, Spain). Cells were cultured at 37 °C with a 5% CO_2_ in high‐glucose Dulbecco's modified Eagle's medium (DMEM) supplemented with 10% (v/v) fetal bovine serum and 100 U·mL^−1^ penicillin and streptomycin, as described [20]. For western blotting, cells (5 × 10^6^) were plated in 100‐mm petri dish and cultured for 48 h, while, for Ca^2+^ imaging cells (4 × 10^5^) were seeded in a 35‐mm six‐well multidish.

### 2.3 Growth conditions for lactobacilli and inactivation

Colonies from *Lacticaseibacillus paracasei* (CECT 9610) and *Lactiplantibacillus plantarum* (CECT 9608) were grown in MRS broth/agar (Oxoid, Dublin, Ireland) at 37 °C in aerobiosis (*Lactiplantibacillus plantarum*) or anaerobiosis (*Lacticaseibacillus paracasei*) for 48 h. Cultures were heat‐inactivated at 80 °C for 2 h and microbial cells were collected after centrifugation at 1036 ***g*** for 10 min. Two washes with sterile water were carried out to remove the remains of the culture medium. Cell pellets were adjusted to a O.D.600 nm (Optical Density) of 1 in sterile water.

### 2.4 Determination of cytosolic free‐Ca^2+^ concentration ([Ca^2+^]_c_)

Cells were loaded with fura‐2 by incubation for 30 min with 2 μm fura‐2/AM at 37 °C as described [33, 34]. Coverslips with cultured cells were mounted on a perfusion chamber and placed on the stage of an epifluorescence inverted microscope (Nikon Eclipse Ti2, Amsterdam, the Netherlands) with an image acquisition and analysis system for videomicroscopy (nis‐elements Imaging Software v.5.02.00; Nikon, Amsterdam, the Netherlands). Cells were superfused at room temperature with HEPES‐buffered saline (HBS) containing 125 mm NaCl, 5 mm KCl, 1 mm MgCl_2_, 5 mm glucose, and 25 mm HEPES, pH 7.4, supplemented with 0.1% (w/v) BSA and examined at 40× magnification (Nikon CFI S FLUOR 40× Oil). Samples were alternatively excited at 340/380 nm as described previously [35]. Fluorescence ratio (F340/F380) was calculated pixel by pixel, and the data were presented as ΔF340/F380. Thapsigargin (TG)‐evoked rises in [Ca^2+^]_c_ and SOCE were estimated as the area under the curve measured as the integral of the rise in fura‐2 fluorescence 340/380 nm ratio during 2.5 min after the addition of the agonist or extracellular Ca^2+^, respectively, and taking a sample every 2 s.

### 2.5 Immunoprecipitation and western blotting

The immunoprecipitation was performed as described previously [36]. Briefly, 500‐μL aliquots of cell suspension (5 × 10^6^ cell·mL^−1^) were lysed. Aliquots of cell lysates (1 mL) were immunoprecipitated by incubation with 2 μg of anti‐FAK antibody and 25 μL of protein A agarose overnight at 4 °C on a rocking platform. The immunoprecipitates were resolved by 10% SDS/PAGE, and separated proteins were electrophoretically transferred onto nitrocellulose membranes for subsequent probing. Western blotting was performed as described previously [29]. Briefly, cells cultured on 100‐mm petri dish (5 × 10^6^ cells) were left untreated or treated with lactobacilli postbiotics, as indicated, and subsequently lysed with ice‐cold NP‐40 buffer pH 8 containing 137 mm of NaCl, 20 mm of Tris, 2 mm of EDTA, 10% glycerol, 1% Nonidet P‐40, 1 mm of Na_3_VO_4_, and complete EDTA‐free protease inhibitor tablets. Cell lysates were resolved by 10% SDS/PAGE and separated proteins were electrophoretically transferred onto nitrocellulose membranes for subsequent probing [37]. Immunodetection of Orai1, Orai2, Orai3, STIM1, STIM2, phosphorylated FAK and β‐actin was achieved by incubation for 1 h with anti‐Orai1, anti‐Orai2, anti‐Orai3, anti‐STIM1, anti‐STIM2, anti‐phosphotyrosine or anti‐β‐actin antibody diluted 1 : 1000 in Tris‐buffered saline with Tween (TBST). To detect the primary antibody, blots were incubated for 1 h with fluorescent goat anti‐rabbit IgG StarBright Blue 700 antibody, horseradish peroxidase‐conjugated goat anti‐rabbit IgG antibody diluted 1 : 10 000 in TBST and then, exposed to enhanced chemiluminescence reagents for 5 min. The antibody binding was assessed with a ChemiDoc Imaging System (Bio‐Rad) and the density of bands was measured using image lab 6.1 Software (Bio‐Rad Laboratories, Madrid, Spain). Data were normalized to the amount of β‐actin from the same gel.

### 2.6 Wound healing assay

The wound healing assay was performed as described previously [33]. Cells were seeded in a 35‐mm six‐well multidish to obtain confluence after 24 h. Next, cells were cultured in medium supplemented with 1% serum, and a wound was created using a sterile 200‐μL plastic pipette tip. Photographs were taken immediately or at the times indicated using an EVOS FL Auto 2 Imaging System (ThermoFisher Scientific). Migration of cells was quantitated using fiji imagej (NIH, Bethesda, MD, USA). Between 15 and 20 measurements were randomly taken along the wound for every single experiment.

### 2.7 Cell viability assay

Cell viability was estimated using the Live/Dead^®^ viability/cytotoxicity kit as described previously [38]. Briefly, cells were incubated for 45 min with 2 μm of calcein‐AM and 4 μm of propidium iodide. Samples were excited at 430 and 555 nm for calcein and propidium iodide, respectively. Fluorescence emission at 542 nm (for viable cells) and 624 nm (for dead cells) was recorded using an EVOS FL auto 2 cell imaging system (ThermoFisher Scientific). Cell viability was quantitated using fiji imagej (NIH).

### 2.8 Statistical analysis

All data are presented as the mean ± standard error of mean (SEM). Analysis of statistical significance was performed using graphpad prism v.9 (GraphPad Software, San Diego, CA, USA). Kruskal–Wallis test combined with Dunn's *post hoc* test were used to compare the different experimental groups. For comparison between two groups, the Mann–Whitney *U* test was used. All data with *P* < 0.05 was deemed significant.

### 2.9 Ethics approval

Experimental procedures were approved by the local ethical committee (University of Extremadura).

## 3 Results

### 3.1 Expression and function of Orai and STIM proteins in the adenocarcinoma cell lines HT‐29 and Caco‐2 and non‐tumoral NCM460 cells

**Fig. 1:**
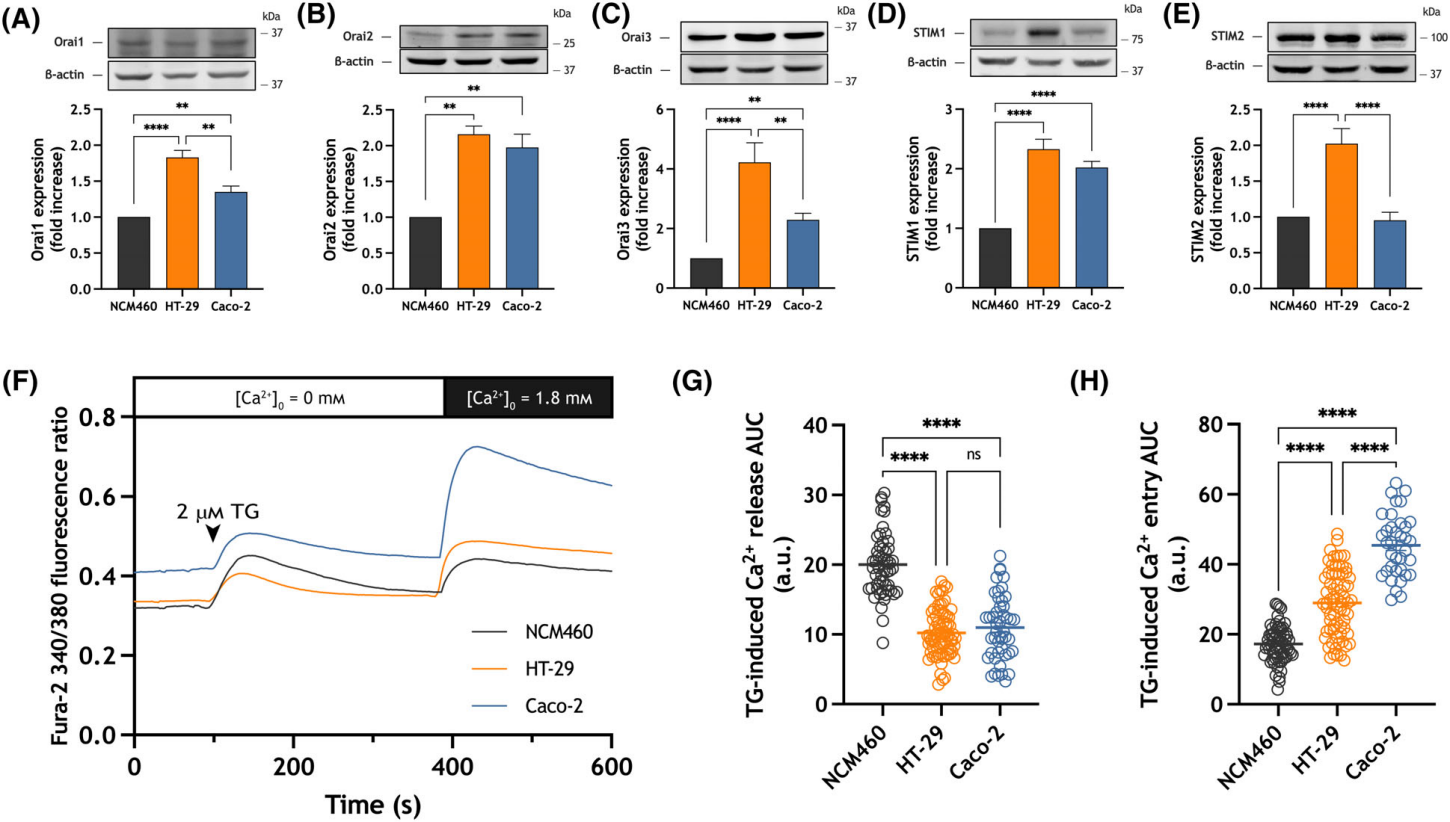
Expression of Orai and stromal interaction molecule (STIM) proteins and Store‐operated Ca^2+^ entry (SOCE) is enhanced in the colorectal adenocarcinoma cell lines HT‐29 and Caco‐2. (A–E) NCM460, HT‐29 and Caco‐2 cells were lysed and the whole cell lysates were analyzed by western blotting using anti‐Orai1 (A), anti‐Orai2 (B), anti‐Orai3 (C), anti‐STIM1 (D) or anti‐STIM2 (E) antibody. Molecular masses indicated on the right were determined using molecular‐mass markers run in the same gel. Membranes were probed with anti‐β‐actin antibody for protein loading control. These results are representative of four separate experiments. Quantification of protein expression in NCM460 (*n* = 4), HT‐29 (*n* = 4) and Caco‐2 (*n* = 4) cells normalized to the β‐actin expression is depicted in the bar graph. Data are represented as mean ± standard error of the mean (SEM) and were statistically analyzed using Kruskal–Wallis test combined with Dunn's *post hoc* test. **P < 0.01 and ****P < 0.0001. (F) Representative Ca^2+^ mobilization in response to 2 μm thapsigargin (TG) measured using fura‐2 in NCM460 (*n* = 3 [62 cells]), HT‐29 (*n* = 3 [69 cells]) and Caco‐2 (*n* = 3 [36 cells]) cells. Cells were superfused with a Ca^2+^‐free Hepes Buffer Saline (HBS) (100 μm ethylene glycol‐bis(2‐aminoethylether)‐*N*,*N*,*N*′,*N*′‐tetraacetic acid (EGTA) added) and stimulated with 2 μm TG, followed by re‐addition of CaCl_2_ (1.8 mm) to estimate Ca^2+^ influx. (G, H) Quantification of TG‐evoked Ca^2+^ release from the intracellular stores and entry in NCM460 (*n* = 3 [62 cells]), HT‐29 (*n* = 3 [69 cells]) and Caco‐2 (*n* = 3 [36 cells]) cells is shown in the scatter plots. Data in bar graphs are represented as mean ± SEM and were statistically analyzed using Kruskal–Wallis test combined with Dunn's *post hoc* test. ****P < 0.0001. a.u., arbitrary units; AUC, area under the curve.

Previous studies have demonstrated that the expression of Orai and STIM family members is altered in colorectal cancer cell lines and patient samples [14, 30, 31]. We have analyzed the expression of Orai and STIM at the protein level in HT‐29 cells and Caco‐2 cells, two of the most widely studied adenocarcinoma cell lines, by western blotting using specific antibodies. As shown in Fig. 1A–E, the expression of Orai1‐3 and STIM1 was significantly higher in HT‐29 and Caco‐2 cells than in NCM460 cells (*P* < 0.01), while that of STIM2 was only overexpressed in HT‐29 cells (*P* < 0.0001).

As shown in Fig. 1F, treatment of NCM460 cells with the SERCA inhibitor TG in the absence of extracellular Ca^2+^ results in a transient increase in [Ca^2+^]_c_, which reveals both Ca^2+^ release from the intracellular stores, mainly the endoplasmic reticulum, as well as Ca^2+^ clearance from the cytosol through the plasma membrane. Subsequent addition of 1.8 mm Ca^2+^ to the extracellular medium led to a sustained increase in [Ca^2+^]_c_ indicative of SOCE. As a result of the upregulation of Orai and STIM isoforms in the adenocarcinoma cell lines, TG‐evoked SOCE was significantly greater in HT‐29 and, especially, in Caco‐2 than in NCM460 (Fig. 1F,H, *P* < 0.0001).

We detected a significant decrease in TG‐induced Ca^2+^ mobilization in the absence of extracellular Ca^2+^ in HT‐29 and Caco‐2 cells (Fig. 1F,G, *P* < 0.0001) probably due to a reduced accumulation of Ca^2+^ into the intracellular stores as previously mentioned [14]. It is also worth mentioning that Caco‐2 cells exhibit an enhanced fura‐2 fluorescence ratio at resting state, which is indicative of a higher basal [Ca^2+^]_c_ than normal mucosa cells or the colorectal HT‐29 cancer cells (Fig. 1F).

### 3.2 Exposure to heat‐killed *Lacticaseibacillus paracasei* and *Lactiplantibacillus plantarum* attenuates SOCE in the adenocarcinoma cell lines HT‐29 and Caco‐2 but not in non‐tumoral NCM460 cells

**Fig. 2:**
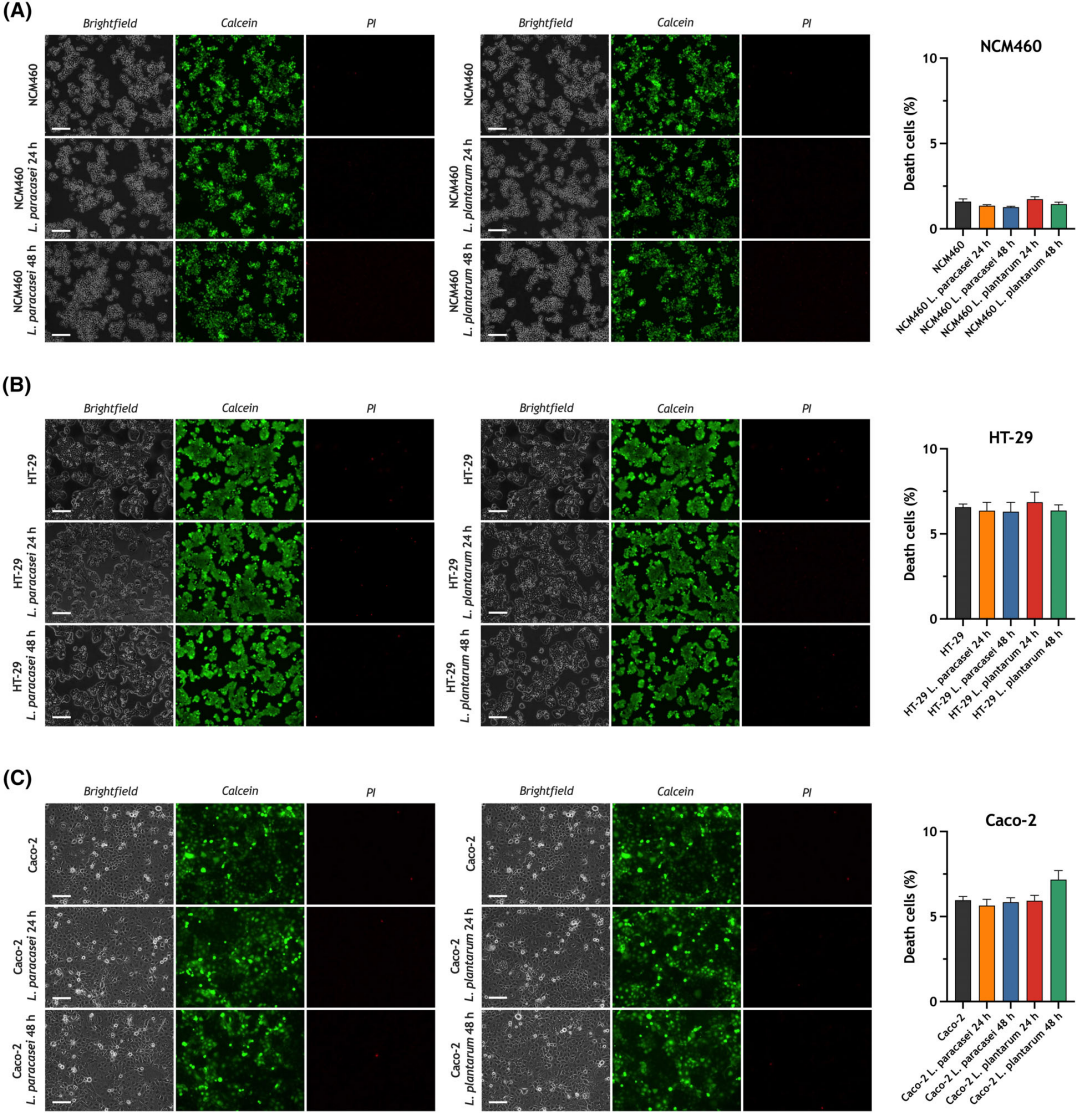
Effect of exposure to heat‐killed *Lacticaseibacillus paracasei* or *Lactiplantibacillus plantarum* on cell viability. NCM460 (A), HT‐29 (B) and Caco‐2 (C) cells were exposed for 24 and 48 h to heat‐killed *Lacticaseibacillus paracasei* or *Lactiplantibacillus plantarum* (10^8^ colony forming units (CFU)·mL^−1^), or left untreated, as indicated. Forty‐eight hours after transfection, cells were loaded with calcein (green) and propidium iodide (PI) (red) and cell staining was visualized using an inverted microscope, as described in Section 2. Bar graphs represent the ratio between PI‐stained cells and total number of cells under the different conditions expressed as fold change experimental over control (untreated cells) and presented as mean ± SEM. Images shown are representative of six independent experiments. Data were statistically analyzed using Kruskal–Wallis test (no significant differences were found). Scale bar: 100 μm.

Previous studies have provided evidence supporting that *Lacticaseibacillus paracasei* and *Lactiplantibacillus plantarum* elicit anti‐colorectal cancer effects [39, 40]. Here we have tested the effect of exposure to heat‐killed *Lacticaseibacillus paracasei* and *Lactiplantibacillus plantarum* on SOCE in the adenocarcinoma cell lines HT‐29 and Caco‐2 as well as in normal colon mucosa NCM460 cells. First, we have investigated whether cell exposure to heat‐killed *Lacticaseibacillus paracasei* and *Lactiplantibacillus plantarum* alters cell viability by using the Live/Dead® viability/cytotoxicity kit. As depicted in Fig. 2, cell viability in cultures of untreated NCM460, HT‐29 and Caco‐2 cells ranges between 95% and 98%. The percentage of viable cells was not significantly modified by cell exposure for 24 or 48 h to heat‐killed *Lacticaseibacillus paracasei* and *Lactiplantibacillus plantarum* (Fig. 2), which indicates that treatment with these postbiotics did not alter cell viability.

**Fig. 3:**
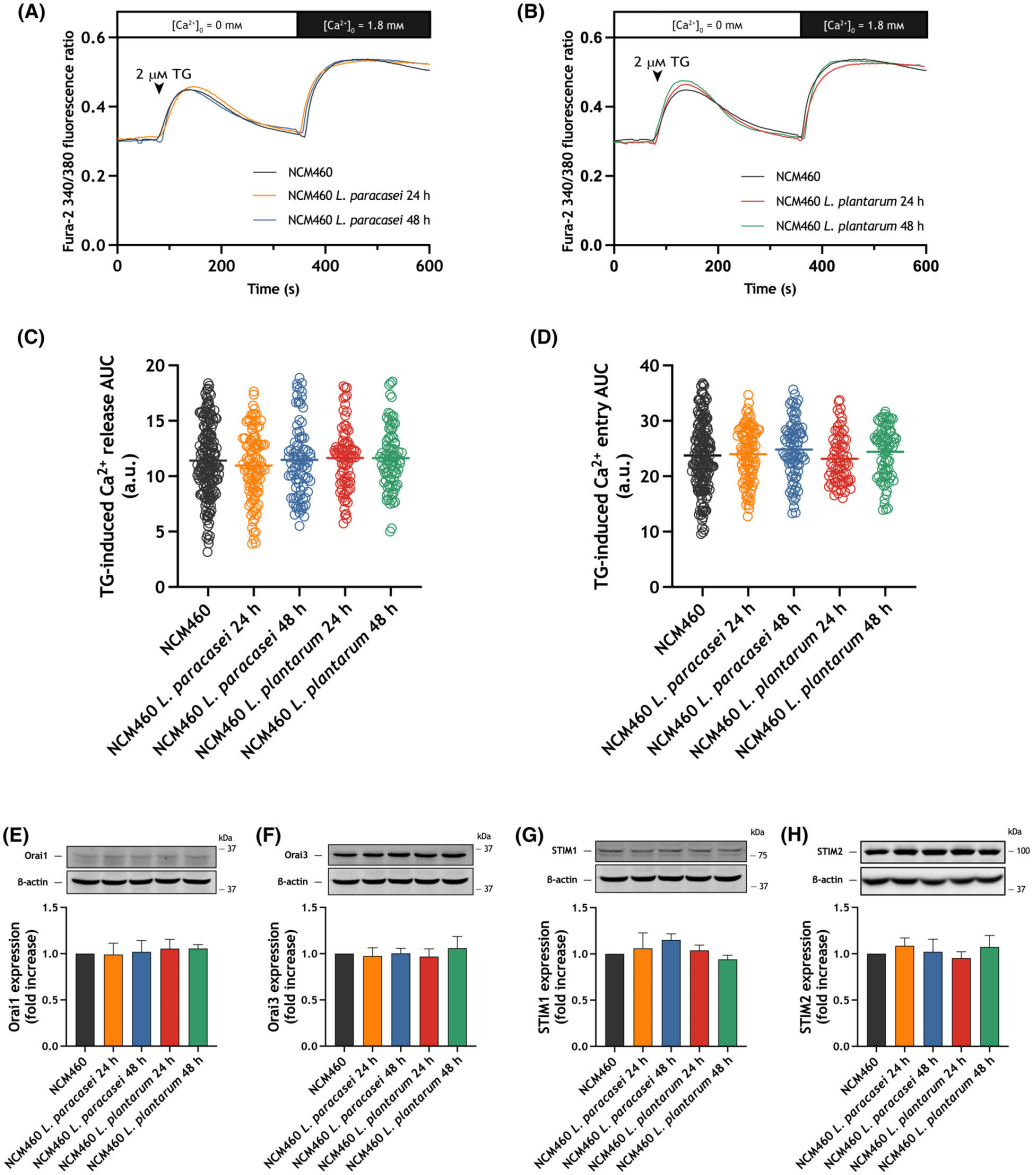
Effect of exposure to heat‐killed *Lacticaseibacillus paracasei* or *Lactiplantibacillus plantarum* on store‐operated Ca^2+^ entry (SOCE) in NCM460 cells. NCM460 cells were exposed for 24 and 48 h to heat‐killed *Lacticaseibacillus paracasei* or *Lactiplantibacillus plantarum* (10^8^ colony forming units (CFU)·mL^−1^). (A, B) Representative Ca^2+^ mobilization in response to 2 μm thapsigargin (TG) measured using fura‐2 in NCM460 cells. Cells were superfused with a Ca^2+^‐free Hepes Buffer Saline (HBS) (100 μm ethylene glycol‐bis(2‐aminoethylether)‐*N*,*N*,*N*′,*N*′‐tetraacetic acid (EGTA) added) and stimulated with 2 μm TG, followed by re‐addition of CaCl_2_ (1.8 mm) to estimate Ca^2+^ influx. Quantification of TG‐evoked Ca^2+^ release from the intracellular stores (C) and entry (D) in non‐exposed (*n* = 4 [150 cells]), 24 h *Lacticaseibacillus paracasei* exposed (*n* = 4 [96 cells]), 48 h *Lacticaseibacillus paracasei* exposed (*n* = 4 [85 cells]), 24 h *Lactiplantibacillus plantarum* exposed (*n* = 4 [83 cells]), 48 h *Lactiplantibacillus plantarum* exposed (*n* = 4 [87 cells]) NCM460 cells is shown in the scatter plots. Data are represented as mean ± standard error of the mean (SEM) and were statistically analyzed using Kruskal–Wallis test (no significant differences were found). (E–H) NCM460 cells were exposed for 24 and 48 h to heat‐killed *Lacticaseibacillus paracasei* or *Lactiplantibacillus plantarum* (10^8^ CFU·mL^−1^), or left untreated, and lysed. The whole cell lysates were analyzed by western blotting using anti‐Orai1 (E), anti‐Orai3 (F), anti‐STIM1 (G) or anti‐STIM2 (H). Molecular masses indicated on the right were determined using molecular‐mass markers run in the same gel. Membranes were probed with anti‐β‐actin antibody for protein loading control. These results are representative of four separate experiments. Quantification of Orai and STIM protein expression normalized to the β‐actin expression is depicted in the bar graphs. Data are represented as mean ± SEM and were statistically analyzed using Kruskal–Wallis test. a.u., arbitrary units; AUC, area under the curve.

Exposure of NCM460 cells to heat‐killed *Lacticaseibacillus paracasei* for 24 and 48 h was unable to modify significantly either Ca^2+^ release or entry (Fig. 3A,C,D), and similar results were obtained when NCM460 cells were treated for 24 and 48 h with heat‐killed *Lactiplantibacillus plantarum* (Fig. 3B–D). We did not detect any modification in the expression of Orai1, Orai3, STIM1 and STIM2 upon treatment of NCM460 cells with heat‐killed *Lacticaseibacillus paracasei* or *Lactiplantibacillus plantarum* (Fig. 3E–H).

**Fig. 4:**
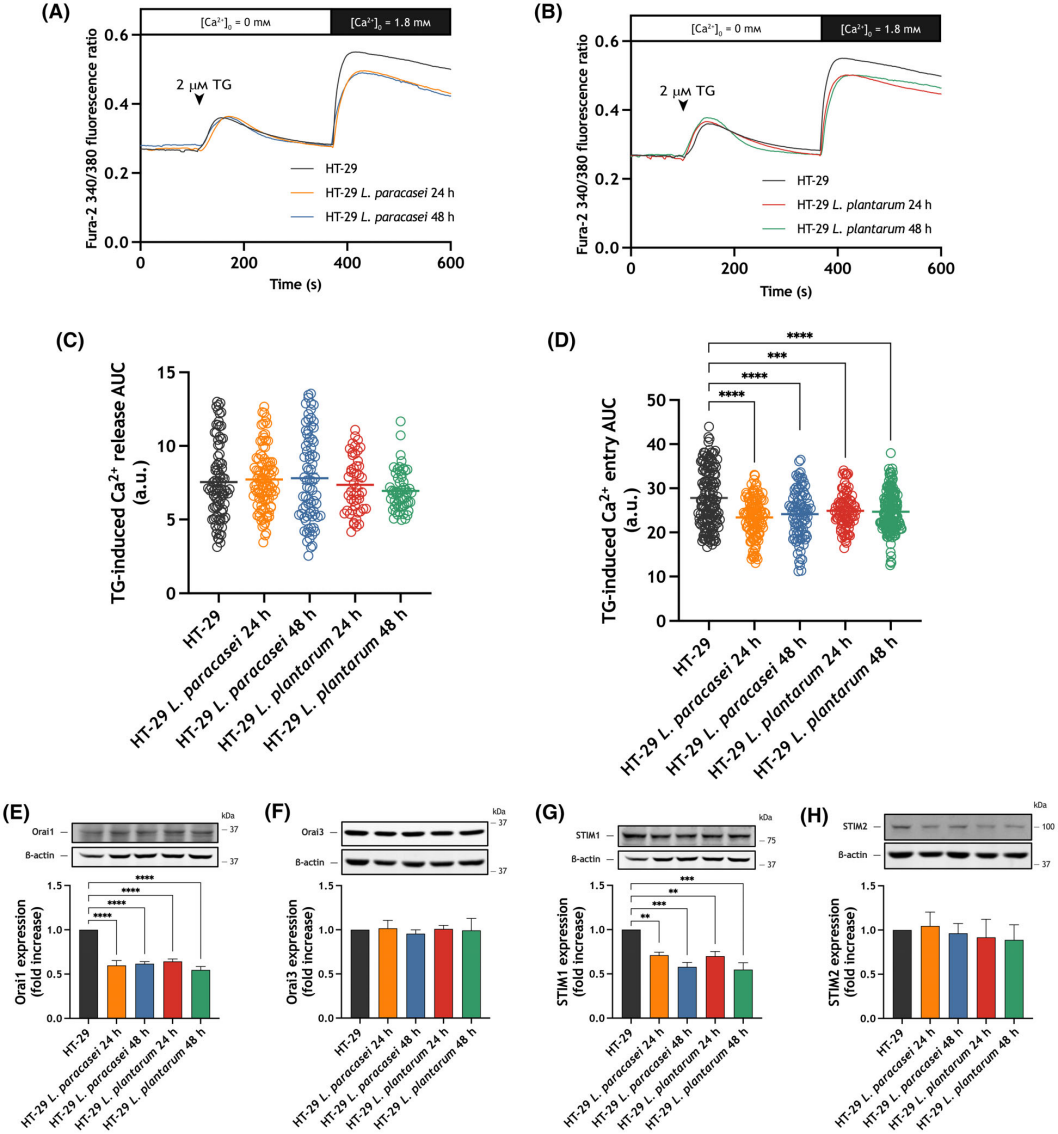
Effect of HT‐29 cell exposure to heat‐killed *Lacticaseibacillus paracasei* or *Lactiplantibacillus plantarum* on store‐operated Ca^2+^ entry (SOCE) and Orai and STIM expression. (A–D) HT‐29 cells were exposed for 24 and 48 h to heat‐killed *Lacticaseibacillus paracasei* or *Lactiplantibacillus plantarum* (10^8^ colony forming units (CFU)·mL^−1^), or left untreated. (A, B) Representative Ca^2+^ mobilization in response to 2 μm thapsigargin (TG) measured using fura‐2 in HT‐29 cells. Cells were superfused with a Ca^2+^‐free Hepes Buffer Saline (HBS) (100 μm ethylene glycol‐bis(2‐aminoethylether)‐*N*,*N*,*N*′,*N*′‐tetraacetic acid (EGTA) added) and stimulated with 2 μm TG, followed by re‐addition of CaCl_2_ (1.8 mm) to estimate Ca^2+^ influx. Quantification of TG‐evoked Ca^2+^ release (C) and entry (D) in non‐exposed (*n* = 4 [120 cells]), 24 h *Lacticaseibacillus paracasei* exposed (*n* = 4 [105 cells]), 48 h *Lacticaseibacillus paracasei* exposed (*n* = 4 [89 cells]), 24 h *Lactiplantibacillus plantarum* exposed (*n* = 4 [87 cells]), 48 h *Lactiplantibacillus plantarum* exposed (*n* = 4 [84 cells]) HT‐29 cells is shown in the scatter plots. Data are represented as mean ± standard error of the mean (SEM) and were statistically analyzed using Kruskal–Wallis test combined with Dunn's *post hoc* test. ***P < 0.001 and ****P < 0.0001. (E‐H) HT‐29 cells were exposed for 24 and 48 h to heat‐killed *Lacticaseibacillus paracasei* or *Lactiplantibacillus plantarum* (10^8^ CFU·mL^−1^), or left untreated, and lysed. The whole cell lysates were analyzed by western blotting using anti‐Orai1 (E), anti‐Orai3 (F), anti‐STIM1 (G) or anti‐STIM2 (H). Molecular masses indicated on the right were determined using molecular‐mass markers run in the same gel. Membranes were probed with anti‐β‐actin antibody for protein loading control. These results are representative of four separate experiments. Quantification of Orai and STIM protein expression normalized to the β‐actin expression is depicted in the bar graphs. Data are represented as mean ± SEM and were statistically analyzed using Kruskal–Wallis test combined with Dunn's *post hoc* test. **P < 0.01, ***P < 0.001 and ****P < 0.0001. a.u., arbitrary units; AUC, area under the curve.

**Fig. 5:**
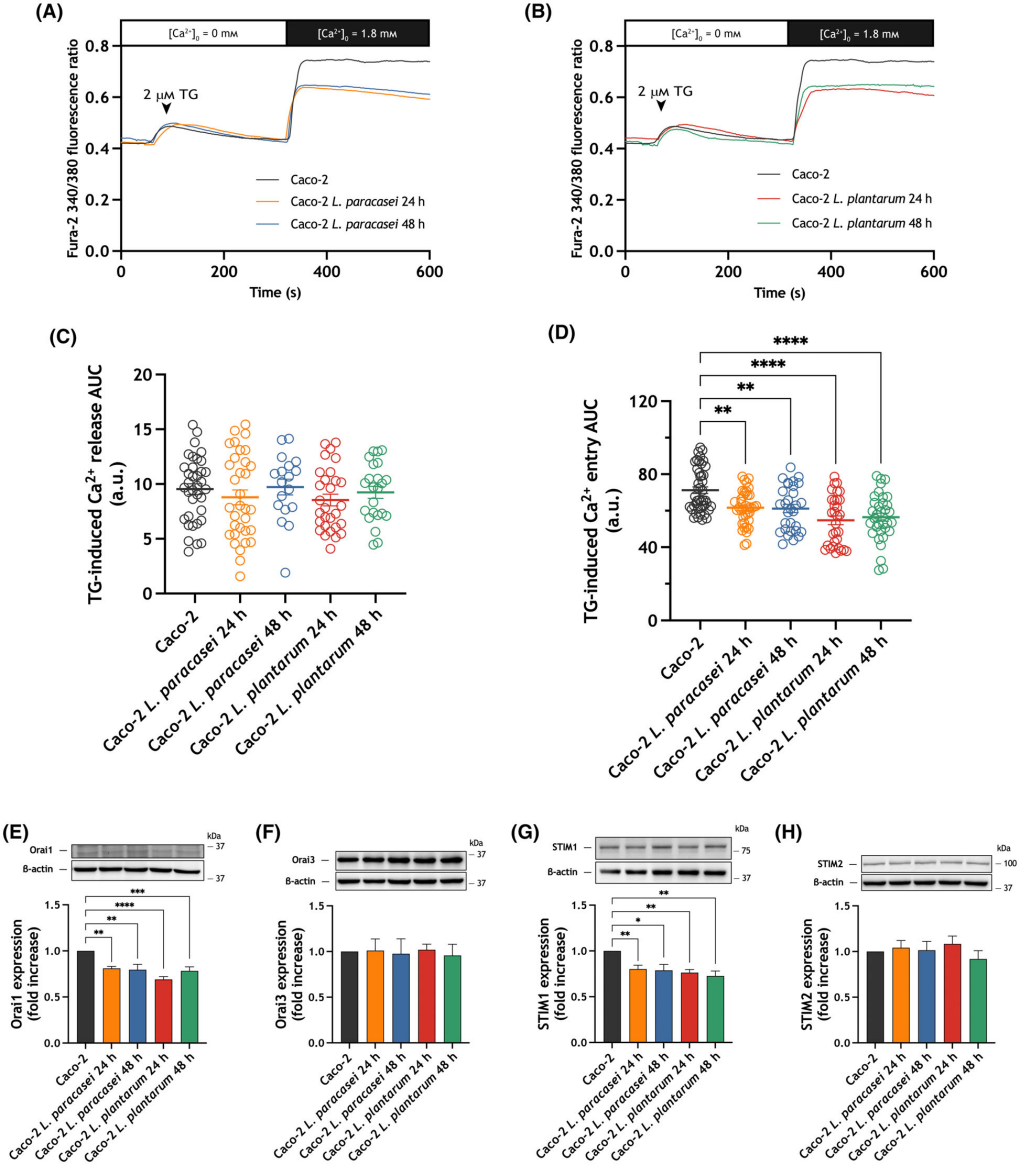
Effect of exposure to heat‐killed *Lacticaseibacillus paracasei* or *Lactiplantibacillus plantarum* on store‐operated Ca^2+^ entry (SOCE) and Orai and STIM expression in Caco‐2 cells. (A–D) Caco‐2 cells were exposed for 24 and 48 h to heat‐killed *Lacticaseibacillus paracasei* or *Lactiplantibacillus plantarum* (10^8^ colony forming units (CFU)·mL^−1^), or left untreated. (A, B) Representative Ca^2+^ mobilization in response to 2 μm thapsigargin (TG) measured using fura‐2 in Caco‐2. Cells were superfused with a Ca^2+^‐free Hepes Buffer Saline (HBS) (100 μm ethylene glycol‐bis(2‐aminoethylether)‐*N*,*N*,*N*′,*N*′‐tetraacetic acid (EGTA) added) and stimulated with 2 μm TG, followed by re‐addition of CaCl_2_ (1.8 mm) to estimate Ca^2+^ influx. Quantification of TG‐evoked Ca^2+^ release (C) and entry (D) in non‐exposed (*n* = 3 [44 cells]), 24 h *Lacticaseibacillus paracasei* exposed (*n* = 3 [39 cells]), 48 h *Lacticaseibacillus paracasei* exposed (*n* = 3 [30 cells]), 24 h *Lactiplantibacillus plantarum* exposed (*n* = 3 [31 cells]), 48 h *Lactiplantibacillus plantarum* exposed (*n* = 3 [34 cells]) Caco‐2 cells is shown in the scatter plots. Data are represented as mean ± standard error of the mean (SEM) and were statistically analyzed using Kruskal–Wallis test combined with Dunn's *post hoc* test. **P < 0.01 and ****P < 0.0001. (E–H) Caco‐2 cells were exposed for 24 and 48 h to heat‐killed *Lacticaseibacillus paracasei* or *Lactiplantibacillus plantarum* (10^8^ CFU·mL^−1^), or left untreated, and lysed. The whole cell lysate was analyzed by western blotting using anti‐Orai1 (E), anti‐Orai3 (F), anti‐STIM1 (G) or anti‐STIM2 (H). Molecular masses indicated on the right were determined using molecular‐mass markers run in the same gel. Membranes were probed with anti‐β‐actin antibody for protein loading control. These results are representative of four separate experiments. Quantification of Orai and STIM protein expression normalized to the β‐actin expression is depicted in the bar graphs. Data are represented as mean ± SEM and were statistically analyzed using Kruskal–Wallis test combined with Dunn's *post hoc* test. *P < 0.05, **P < 0.01, ***P < 0.001 and ****P < 0.0001. a.u., arbitrary units; AUC, area under the curve.

Interestingly, SOCE was attenuated in HT‐29 cells after 24 and 48 h treatment with heat‐killed *Lacticaseibacillus paracasei*, without having any effect on Ca^2+^ release from the intracellular stores (Fig. 4A,C,D). To elucidate the mechanism underlying the inhibition of SOCE we analyzed the effect of treatment with heat‐killed *Lacticaseibacillus paracasei* on the Orai and STIM expression level. Concerning the expression of Orai proteins we focused our studies on Orai1, which plays a predominant role in the CRAC channels [26, 27], and Orai3, which has been suggested to be an oncogene [41]. Consistent with the effect on SOCE, when HT‐29 cells were exposed to heat‐killed *Lacticaseibacillus paracasei* we detected a significant reduction in Orai1 and STIM1 expression (Fig. 4E,G; *P* < 0.01). No changes in Orai3 and STIM2 expression were detected after treatment with heat‐killed *Lacticaseibacillus paracasei* at the times investigated (Fig. 4F,H). As shown in Fig. 4E–H, treatment of HT‐29 with heat‐killed *Lactiplantibacillus plantarum* induced a sustained decrease in Orai1 and STIM1 expression, at least, for 48 h, with no effect on the Orai3 and STIM2 expression. Consistent with the effect on STIM1 and Orai1 expression, exposure to heat‐killed *Lactiplantibacillus plantarum* for 24 and 48 h significantly attenuated SOCE in HT‐29 cells without having any significant effect on Ca^2+^ release from the intracellular stores (Fig. 4B–D; *P* < 0.001). Similar results were observed when Caco‐2 cells were exposed to heat‐killed *Lacticaseibacillus paracasei* and *Lactiplantibacillus plantarum* (Fig. 5). Altogether, our results indicate that *Lacticaseibacillus paracasei* and *Lactiplantibacillus plantarum* postbiotics attenuate SOCE by reducing Orai1 and STIM1 expression in adenocarcinoma cell lines without having any significant effect on SOCE in normal colon mucosa cells.

### 3.3 Exposure to heat‐killed *Lacticaseibacillus paracasei* and *Lactiplantibacillus plantarum* attenuates the ability of the adenocarcinoma cell lines HT‐29 and Caco‐2 to migrate

**Fig. 6:**
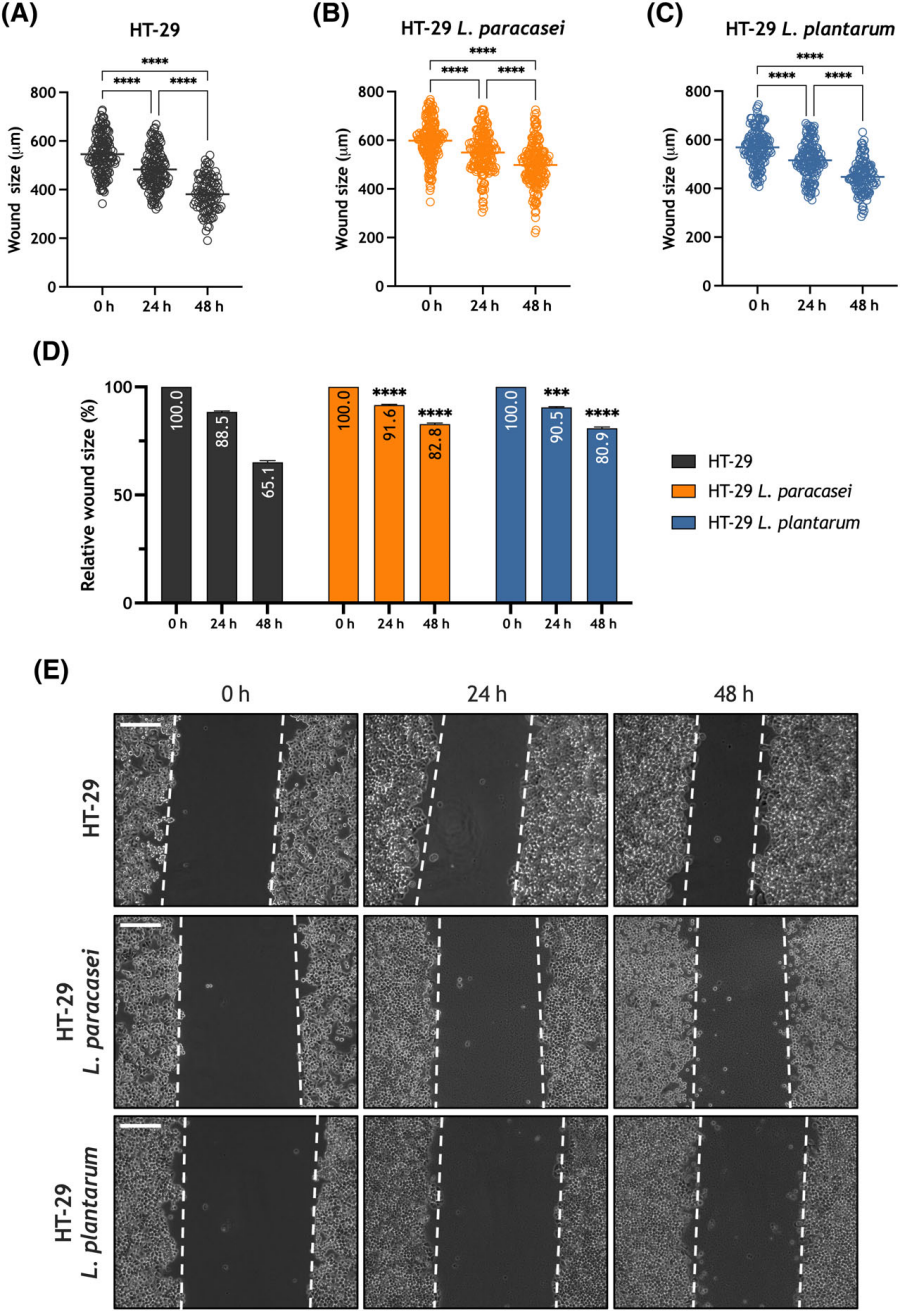
Exposure to heat‐killed *Lacticaseibacillus paracasei* or *Lactiplantibacillus plantarum* attenuates migration of the colorectal adenocarcinoma cell line HT‐29. HT‐29 cells were exposed to heat‐killed *Lacticaseibacillus paracasei* or *Lactiplantibacillus plantarum* (10^8^ colony forming units (CFU)·mL^−1^), as indicated, and were subjected to the wound healing assay, as described in Section 2. (A–C) Quantification of the wound size, in micrometers, in the different conditions: non‐exposed HT‐29 cells (*t* = 0, *n* = 6 [120 measurements]); (*t* = 24, *n* = 6 [120 measurements]); (*t* = 48, *n* = 6 [120 measurements]), *Lacticaseibacillus paracasei* exposed HT‐29 cells (*t* = 0, *n* = 6 [120 measurements]); (*t* = 24, *n* = 6 [120 measurements]); (*t* = 48, *n* = 6 [120 measurements]) and *Lactiplantibacillus plantarum* exposed HT‐29 cells (*t* = 0, *n* = 6 [120 measurements]); (*t* = 24, *n* = 6 [120 measurements]); (*t* = 48, *n* = 6 [120 measurements]). Scatter plots are represented as mean ± standard error of the mean (SEM) and were statistically analyzed using Kruskal–Wallis test with multiple comparisons (Dunn's test). ****P < 0.0001. (D) The bar graph represents the relative wound size upon the different experimental conditions expressed as percentage of the size at time = 0 h and presented as mean ± SEM (non‐exposed HT‐29 cells (*t* = 0, *n* = 6 [120 measurements]); (*t* = 24, *n* = 6 [120 measurements]); (*t* = 48, *n* = 6 [120 measurements]), *Lacticaseibacillus paracasei* exposed HT‐29 cells (*t* = 0, *n* = 6 [120 measurements]); (*t* = 24, *n* = 6 [120 measurements]); (*t* = 48, *n* = 6 [120 measurements]) and *Lactiplantibacillus plantarum* exposed HT‐29 cells (*t* = 0, *n* = 6 [120 measurements]); (*t* = 24, *n* = 6 [120 measurements]); (*t* = 48, *n* = 6 [120 measurements])). Data were statistically analyzed using Kruskal–Wallis test combined with Dunn's *post hoc* test (***P < 0.001 and ****P < 0.0001 as compared to the wound size at the corresponding time in the absence of postbiotics). (E) Images were acquired at 0, 24 and 48 h from the beginning of the assay. Images are presentative of six independent experiments (a total of 120 measurements) for each experimental condition. The dotted lines define the areas lacking cells Scale bar: 200 μm.

**Fig. 7:**
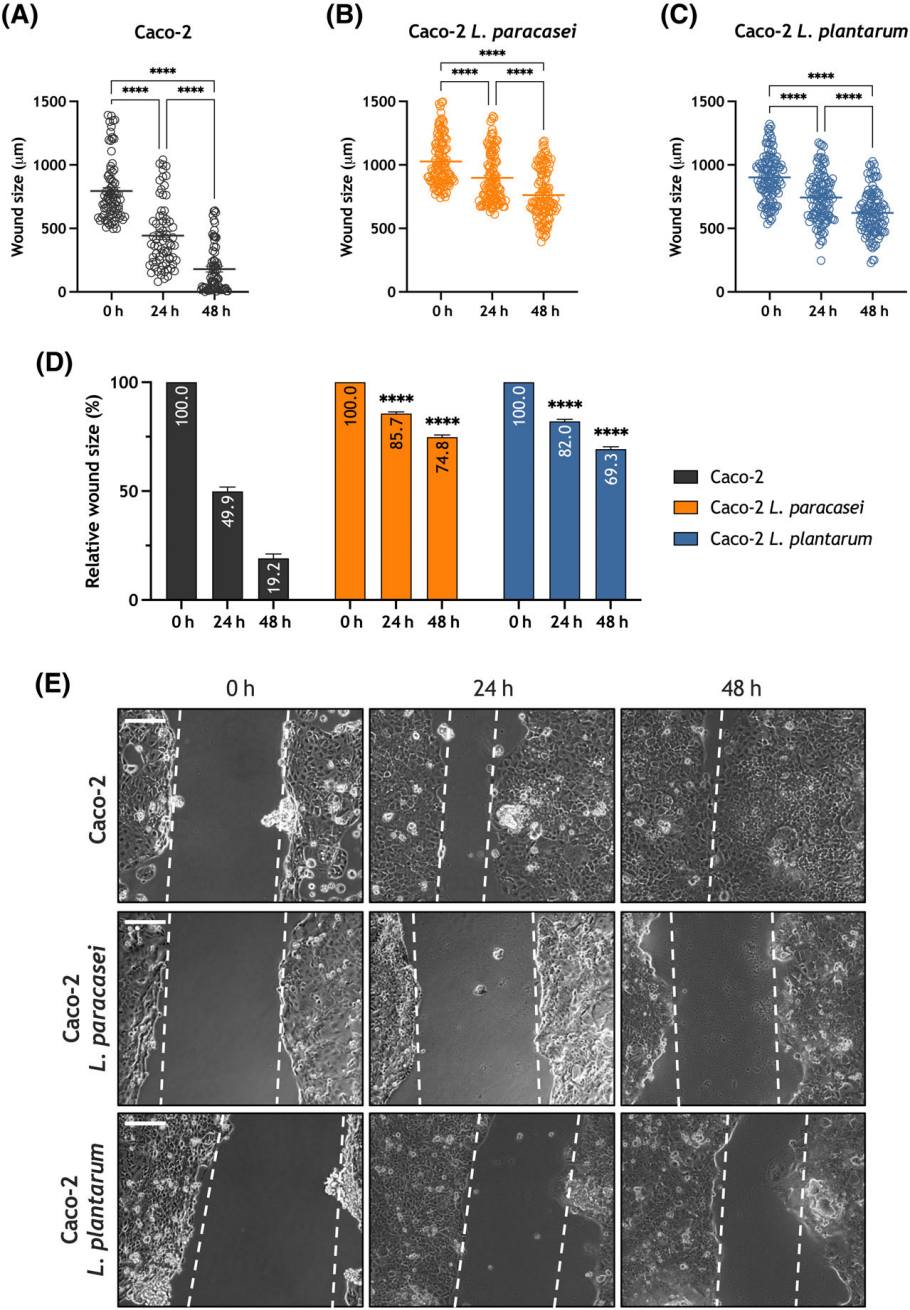
Exposure to heat‐killed *Lacticaseibacillus paracasei* or *Lactiplantibacillus plantarum* attenuates migration of the colorectal adenocarcinoma cell line Caco‐2. Caco‐2 cells were exposed to heat‐killed *Lacticaseibacillus paracasei* or *Lactiplantibacillus plantarum* (10^8^ colony forming units (CFU)·mL^−1^), as indicated, and were subjected to the wound healing assay, as described in Section 2. (A–C) Quantification of the wound size, in micrometers, in the different conditions: non‐exposed Caco‐2 cells (*t* = 0, *n* = 6 [90 measurements]); (*t* = 24, *n* = 6 [90 measurements]); (*t* = 48, *n* = 6 [90 measurements]), *Lacticaseibacillus paracasei* exposed Caco‐2 cells (*t* = 0, *n* = 6 [108 measurements]); (*t* = 24, *n* = 6 [108 measurements]); (*t* = 48, *n* = 6 [108 measurements]) and *Lactiplantibacillus plantarum* exposed Caco‐2 cells (*t* = 0, *n* = 6 [114 measurements]); (*t* = 24, *n* = 6 [114 measurements]); (*t* = 48, *n* = 6 [114 measurements]). Scatter plots are represented as mean ± standard error of the mean (SEM) and were statistically analyzed using Kruskal–Wallis test with multiple comparisons (Dunn's test). ****P < 0.0001. (D) The bar graph represents the relative wound size upon the different experimental conditions expressed as percentage of the size at time = 0 h and presented as mean ± SEM (non‐exposed Caco‐2 cells (*t* = 0, *n* = 6 [90 measurements]); (*t* = 24, *n* = 6 [90 measurements]); (*t* = 48, *n* = 6 [90 measurements]), *Lacticaseibacillus paracasei* exposed Caco‐2 cells (*t* = 0, *n* = 6 [108 measurements]); (*t* = 24, *n* = 6 [108 measurements]); (*t* = 48, *n* = 6 [108 measurements]) and *Lactiplantibacillus plantarum* exposed Caco‐2 cells (*t* = 0, *n* = 6 [114 measurements]); (*t* = 24, *n* = 6 [114 measurements]); (*t* = 48, *n* = 6 [114 measurements])). Data were statistically analyzed using Kruskal–Wallis test combined with Dunn's *post hoc* test (****P < 0.0001 as compared to the wound size at the corresponding time in the absence of postbiotics). (E) Images were acquired at 0, 24 and 48 h from the beginning of the assay. Images are presentative of six independent experiments for each experimental condition with a total measurement of 90, 108 and 114 in non‐exposed, *Lacticaseibacillus paracasei* exposed and *Lactiplantibacillus plantarum* exposed Caco‐2 cells. respectively. The dotted lines define the areas lacking cells. Scale bar: 200 μm.

Next, we assessed the relevance of exposure to heat‐killed *Lacticaseibacillus paracasei* and *Lactiplantibacillus plantarum* in the ability of the adenocarcinoma cell lines HT‐29 and Caco‐2 to migrate. HT‐29 and Caco‐2 cells were subjected to the well‐established wound healing assay. Cells were seeded, scratched, and cultured in medium supplemented with 1% serum to prevent cell growth. Cell migration was estimated as described in Section 2. As shown in Fig. 6A,D,E, untreated HT‐29 cells reduced the wound size during the first 48 h (*P* < 0.0001; *n* = 6). Exposure to heat‐killed *Lacticaseibacillus paracasei* significantly attenuated the ability of HT‐29 cells to migrate (Fig. 6B,D,E; *P* < 0.0001; *n* = 6), despite we still found a significant reduction in the wound size (Fig. 6B; *P* < 0.0001). Similarly, exposure to heat‐killed *Lactiplantibacillus plantarum* significantly reduced HT‐29 cell migration (Fig. 6C–E; *P* < 0.0001; *n* = 6). Similar effects were observed when we assessed the effect of exposure to heat‐killed *Lacticaseibacillus paracasei* and *Lactiplantibacillus plantarum* on Caco‐2 cell migration. As shown in Fig. 7A,D,E, these cells exhibit a greater ability to migrate than HT‐29 cells, as a result, after 48 h the wound was completely healed in a significant number of determinations (*P* < 0.0001; *n* = 6). Exposure to heat‐killed *Lacticaseibacillus paracasei* or and *Lactiplantibacillus plantarum* significantly attenuated the ability of Caco‐2 cells to migrate (Fig. 7B–E; *P* < 0.0001; *n* = 6), despite we still were able to detect a significant reduction in the wound size during the first 48 h (Fig. 7B–E; *P* < 0.0001). Altogether, these findings indicate that exposure or the adenocarcinoma HT‐29 and Caco‐2 cells to heat‐killed *Lacticaseibacillus paracasei* and *Lactiplantibacillus plantarum* attenuates their ability to migrate.

**Fig. 8:**
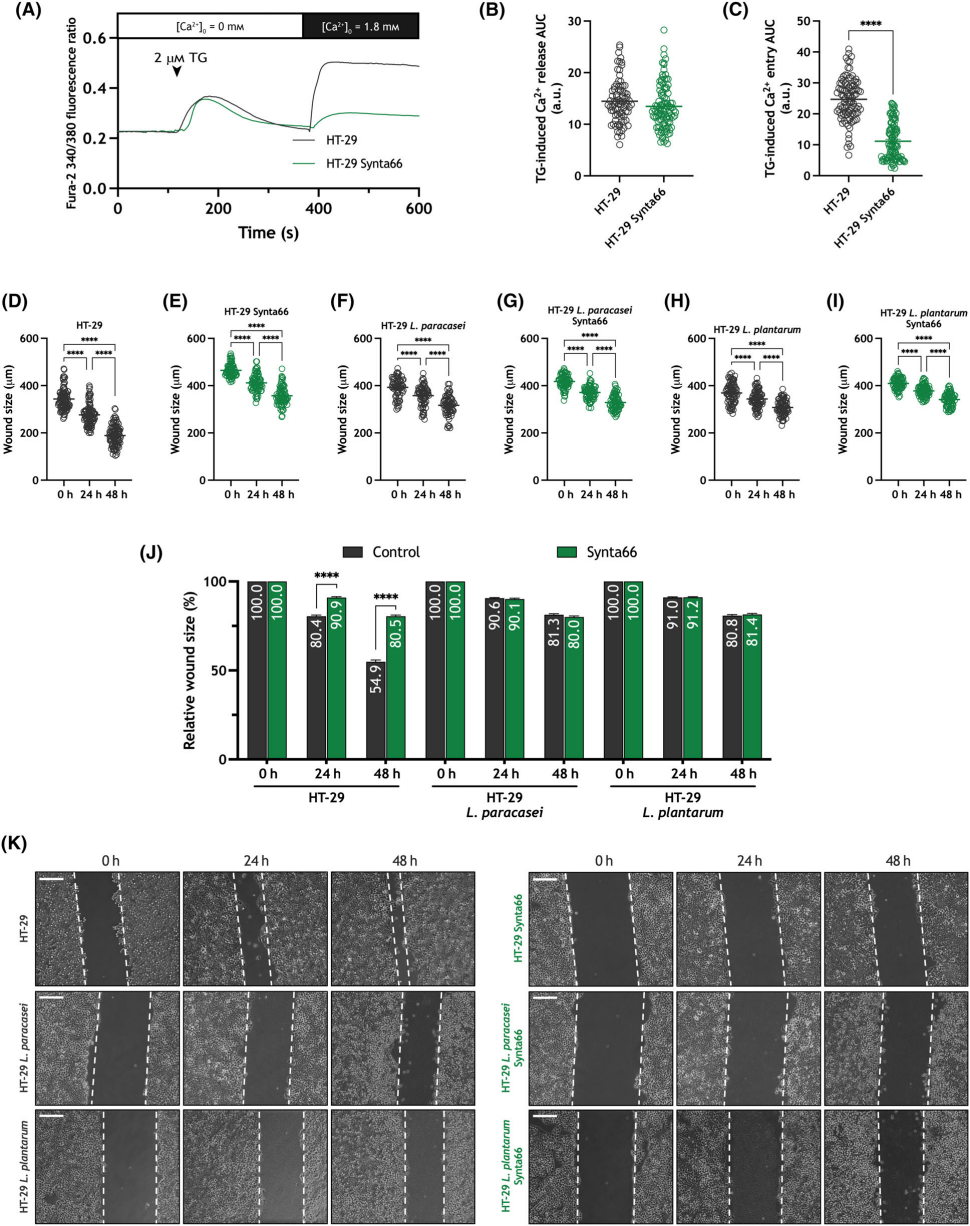
Synta66 inhibits store‐operated Ca^2+^ entry (SOCE) and migration in the colorectal adenocarcinoma cell line HT‐29. (A–C) Fura‐2‐loaded HT‐29 cells were treated with 1 μm synta66 or the vehicle for 5 min, as indicated. Cells were superfused with a Ca‐free Hepes Buffer Saline (HBS) (100 μm ethylene glycol‐bis(2‐aminoethylether)‐*N*,*N*,*N*′,*N*′‐tetraacetic acid (EGTA) added) and stimulated with 2 μm thapsigargin (TG), followed by re‐addition of CaCl_2_ (1.8 mm) to estimate Ca^2+^ influx. Quantification of TG‐evoked Ca^2+^ release (B) and entry (C) in untreated (*n* = 4 [100 cells]) and synta66 treated (*n* = 4 [98 cells]) HT‐29 cells is shown in the scatter plots. Data are represented as mean ± standard error of the mean (SEM) and were statistically analyzed using the Mann–Whitney *U* test. ****P < 0.0001. (D–K) HT‐29 cells were treated with 1 μm synta66 or the vehicle and exposed to heat‐killed *Lacticaseibacillus paracasei* or *Lactiplantibacillus plantarum* (10^8^ colony forming units (CFU)·mL^−1^), or left untreated, as indicated. Cells were subjected to the wound healing assay, as described in Section 2. (D–I) Quantification of the wound size, in micrometers, in the different conditions: non‐exposed and synta66 untreated HT‐29 cells (*t* = 0, *t* = 24, *t* = 48; *n* = 6 [100 measurements]), non‐exposed and synta66 treated HT‐29 cells (*t* = 0, *t* = 24 *t* = 48; *n* = 6 [90 measurements]), *Lacticaseibacillus paracasei* exposed and untreated HT‐29 cells (*t* = 0, *t* = 24, *t* = 48; *n* = 6 [90 measurements]), *Lacticaseibacillus paracasei* exposed and synta66 treated HT‐29 cells (*t* = 0, *t* = 24, *t* = 48; *n* = 6 [90 measurements]), *Lactiplantibacillus plantarum* exposed and untreated HT‐29 cells (*t* = 0, *t* = 24, *t* = 48; *n* = 6 [90 measurements]), *Lactiplantibacillus plantarum* exposed and synta66 treated HT‐29 cells (*t* = 0, *t* = 24, *t* = 48; *n* = 6 [90 measurements]). Scatter plots are represented as mean ± standard error of the mean (SEM) and were statistically analyzed using Kruskal–Wallis test with multiple comparisons (Dunn's test). ****P < 0.0001. (J) The bar graph represents the relative wound size upon the different experimental conditions expressed as percentage of the size at time = 0 h and presented as mean ± SEM (six independent experiments for each experimental condition with a total measurement of 100 in non‐exposed and synta66 untreated HT‐29 cells, 90 in non‐exposed and synta66 treated HT‐29 cells, *Lacticaseibacillus paracasei* exposed and untreated HT‐29 cells, *Lacticaseibacillus paracasei* exposed and synta66 treated HT‐29 cells, *Lactiplantibacillus plantarum* exposed and untreated HT‐29 cells and *Lactiplantibacillus plantarum* exposed and synta66 treated HT‐29). Data were statistically analyzed using Kruskal–Wallis test combined with Dunn's *post hoc* test (****P < 0.0001 as compared to the wound size at the corresponding time in the absence of synta66). (K) Images were acquired at 0, 24 and 48 h from the beginning of the assay. Images are presentative of six independent experiments for each experimental condition with a total measurement of 100 in non‐exposed and synta66 untreated HT‐29 cells, 90 in the rest of conditions. The dotted lines define the areas lacking cells. Scale bar: 200 μm. a.u., arbitrary units; AUC, area under the curve.

As treatment of adenocarcinoma cells with heat‐killed *Lacticaseibacillus paracasei* and *Lactiplantibacillus plantarum* attenuates both SOCE and cell migration we can assume that the latter is a consequence of the inhibition of SOCE. To further test this possibility, we have used the Orai specific inhibitor synta66 [42]. We noticed that treatment of HT‐29 cells with 1 μm synta66 for 5 min almost completely inhibited SOCE (Fig. 8A–C; *P* < 0.0001), without having any significant effect on TG‐evoked Ca^2+^ release from internal stores. Treatment with synta66 significantly reduced the ability of HT‐29 cells to migrate (Fig. 8D,E,J; *P* < 0.0001), thus indicating that SOCE plays a relevant role in cell migration. Interestingly, in the presence of synta66 cell exposure to heat‐killed *Lacticaseibacillus paracasei* and *Lactiplantibacillus plantarum* did not induce any further inhibition of HT‐29 cell migration (Fig. 8F–I). These findings strongly suggest that the effect of *Lacticaseibacillus paracasei* and *Lactiplantibacillus plantarum* postbiotics are entirely mediated by inhibition of SOCE.

### 3.4 Treatment of HT‐29 and Caco‐2 cells with heat‐killed *Lacticaseibacillus paracasei* or *Lactiplantibacillus plantarum* attenuates FAK tyrosine phosphorylation

**Fig. 9:**
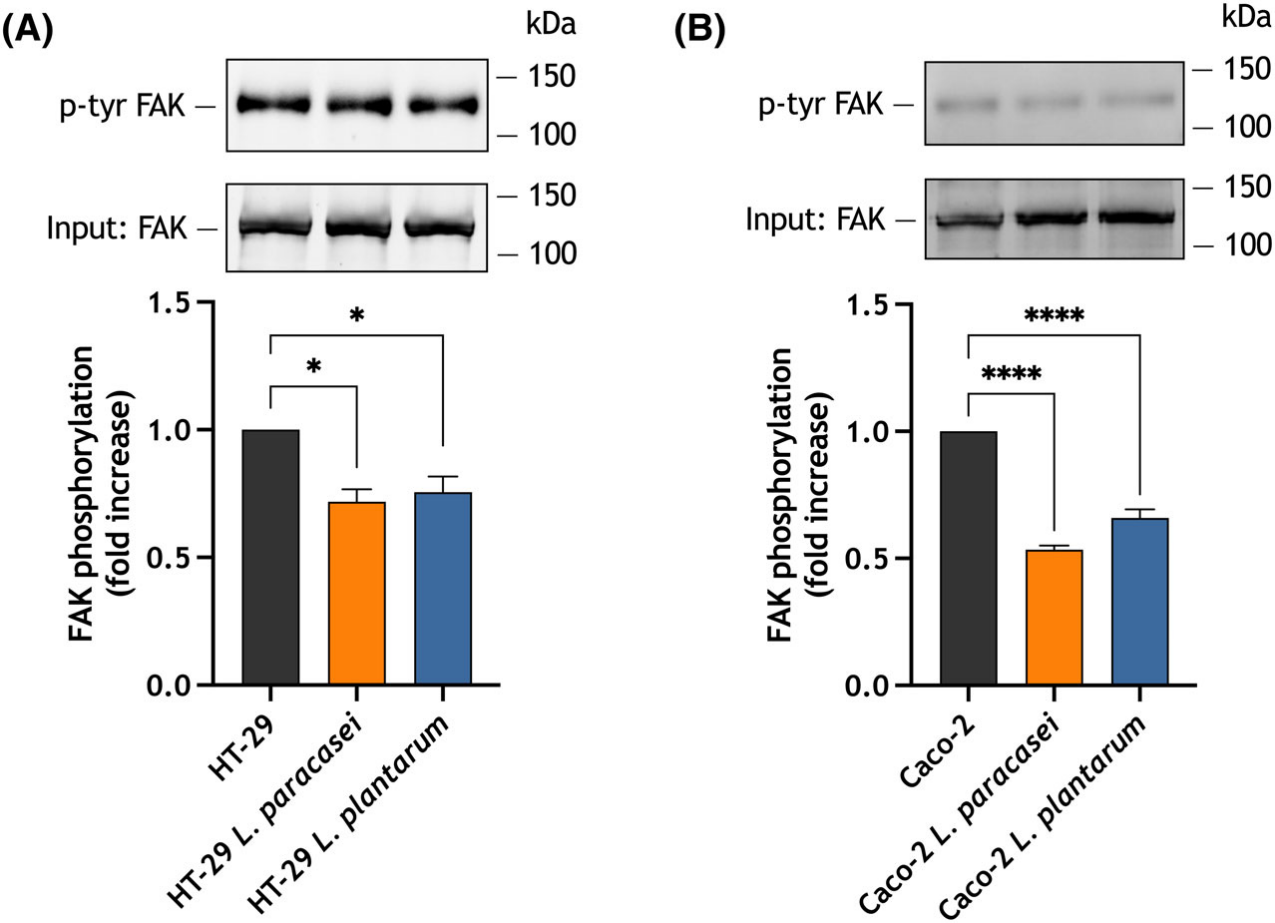
Effect of exposure to heat‐killed *Lacticaseibacillus paracasei* or *Lactiplantibacillus plantarum* on focal adhesion kinase (FAK) tyrosine phosphorylation in the colorectal adenocarcinoma cell lines HT‐29 and Caco‐2. HT‐29 (A) and Caco‐2 cells (B) were exposed for 24 h to heat‐killed *Lacticaseibacillus paracasei* or *Lactiplantibacillus plantarum* (10^8^ colony forming units (CFU)·mL^−1^), as indicated, and lysed. Whole cell lysates were immunoprecipitated with anti‐FAK antibody. Immunoprecipitates were analyzed by SDS/PAGE and western blotting with anti‐phosphotyrosine monoclonal antibody (4G10) or anti‐FAK antibody. Membranes were reprobed with anti‐FAK antibody for protein loading control. Blots are representative of four separate experiments. The bar graphs represent FAK tyrosine phosphorylation presented as the phospho‐FAK/total FAK ratio. Data are represented as mean ± standard error of the mean (SEM) and were statistically analyzed using Kruskal–Wallis test combined with Dunn's *post hoc* test. *P < 0.05 and ****P < 0.0001 as compared to FAK tyrosine phosphorylation in the absence of postbiotics.

Phosphorylation and subsequent activation of the cytosolic focal adhesion kinase (FAK) has been reported to play an important role in cell migration and invasion. FAK activity is essential for the formation of focal adhesions, which, in turn, are crucial for cell migration [43]. We have previously reported that FAK tyrosine phosphorylation and activation is modulated by SOCE and, more precisely, Orai1 [44, 45]. Hence, we have further explored whether FAK phosphorylation is a target of heat‐killed *Lacticaseibacillus paracasei* and *Lactiplantibacillus plantarum* and underlies the attenuation of migration in the colorectal adenocarcinoma cell lines HT‐29 and Caco‐2. As depicted in Fig. 9, exposure of HT‐29 and Caco‐2 cells to heat‐killed *Lacticaseibacillus paracasei* and *Lactiplantibacillus plantarum* for 24 h significantly inhibited FAK tyrosine phosphorylation. These observations indicate that treatment of colorectal adenocarcinoma cells with heat‐killed *Lacticaseibacillus paracasei* and *Lactiplantibacillus plantarum* attenuates FAK tyrosine phosphorylation and activation, which might underlie their inhibitory role on cell migration.

## 4 Discussion

Our results indicate that treatment of the colorectal adenocarcinoma cell lines HT‐29 and Caco‐2 with inanimate *Lacticaseibacillus paracasei* and *Lactiplantibacillus plantarum* results in a significant attenuation of SOCE due to downregulation of Orai1 and STIM1 expression. We noticed that colorectal cancer cells exhibit a reduced Ca^2+^ release from the intracellular stores upon stimulation with TG but enhanced SOCE, which is consistent with the findings reported by Sobradillo and coworkers [14]. STIM1 and Orai1 are the key molecular players of SOCE [26, 46] and attenuation of their expression is consistent with the reduced SOCE in the presence of postbiotics. The expression of both proteins is enhanced in CRC cells [12], playing a relevant role in the development of cancer hallmarks [47]. By contrast, our results indicate that treatment of the colorectal adenocarcinoma cell lines with postbiotics of *Lacticaseibacillus paracasei* and *Lactiplantibacillus plantarum* did not significantly alter the protein content of Orai3 and STIM2. Orai3 has been presented as an oncogene and plays an important role in supporting a number of hallmarks in different tumoral cell types, such as pancreatic adenocarcinoma, prostate and luminal breast cancer cells [48, 49, 50, 51]. In CRC cells Orai3 has been reported to be upregulated [14] and is involved in tumor progression [52]. On the other hand, CRC cancer cells exhibit downregulated expression of STIM2 leading to a greater STIM1:STIM2 ratio that supports enhanced SOCE [14]. The lack of effect of the postbiotics on the Orai3 and STIM2 expression indicates that these compounds are not general inhibitors of protein synthesis but selectively reduce Orai1 and STIM1 protein content, either by attenuating their synthesis, enhancing their degradation or both.

Cell incubation with heat‐killed *Lacticaseibacillus paracasei* and *Lactiplantibacillus plantarum* impairs FAK tyrosine phosphorylation and activation leading to inhibited *in vitro* migration. Our results indicate that pharmacological inhibition of SOCE using synta66 significantly reduces CRC cell migration and prevent any further migration inhibition by *Lacticaseibacillus paracasei* and *Lactiplantibacillus plantarum* postbiotics. Furthermore, SOCE has been reported to be involved in focal adhesion turnover [53] and impairment of Orai1 inactivation by adenylyl cyclase 8 plays enhances FAK phosphorylation and activation in triple‐negative breast cancer cells [45]. The antitumoral effects of postbiotics on CRC is consistent with previous studies reporting biological functions of postbiotics against CRC. However, to our knowledge, this is the first description of the role of postbiotics in the regulation of SOCE and associated functions. Metabolites from *Lactiplantibacillus plantarum* have been reported to induced cytotoxic and anti‐proliferative effects in colorectal, breast, cervical, liver and leukemia cancer cell lines [54]. Furthermore, postbiotics of *Lactiplantibacillus plantarum*, *Bifidobacterium bifidum*, *Limosilactobacillus reuteri*, and *Lacticaseibacillus rhamnosus* have been shown to induce apoptosis in MCF‐7 breast cancer cells and gastric cancer MKN1 cells [54, 55]. In addition, postbiotics of *Lacticaseibacillus casei* and *Lacticaseibacillus rhamnosus GG* have been reported to attenuate CRC cell invasion [9]. The molecular mechanisms associated to the antitumoral effects of postbiotics include the interaction of short‐chain fatty acids with G protein‐coupled receptors, which has been reported to underlie the inhibition of MMP‐9, which prevents tissue invasion, downregulation of neuropilin‐1, leading to the impairment of angiogenesis and metastasis of CRC cells or enhancement of Bcl‐2‐associated X protein (Bax) expression, which eventually induces apoptosis [9, 56, 57]. In addition, exopolysaccharides are recognized by the pattern recognition receptors of immune cells resulting in the activation of Syk and, subsequently, NF‐κB, leading to a variety of immune responses [58].

Our results indicate that cell treatment with heat‐killed *Lacticaseibacillus paracasei* and *Lactiplantibacillus plantarum* exerted a sustained inhibition of SOCE and cell migration, at least for the first 48 h of exposure. However, both postbiotics have a negligible effect, if any, on cell viability. More interesting is the fact that while inanimate *Lacticaseibacillus paracasei* and *Lactiplantibacillus plantarum* attenuate SOCE in both colorectal adenocarcinoma cell lines no detectable effects have been observed in normal colon mucosa cells. This selectivity reveals the potential antitumor effect of *Lacticaseibacillus paracasei* and *Lactiplantibacillus plantarum* postbiotics against CRC. Whether preparations of inanimate *Lacticaseibacillus paracasei* and *Lactiplantibacillus plantarum* exert antitumoral effects against other cancer types deserves further studies.

## 5 Conclusions

Summarizing our results indicate that preparations of inanimate *Lacticaseibacillus paracasei* and *Lactiplantibacillus plantarum* selectively impair *in vitro* migration of the colorectal adenocarcinoma cell lines HT‐29 and Caco‐2 by attenuating Orai1 and STIM1 expression and leading to inhibition of Ca^2+^ influx through SOCE and, subsequent, FAK activation.

## Conflict of interest

The authors declare no conflict of interest.

## Author contributions

JAR, JJL and RC conceived and designed research; AM‐D performed experiments; AM‐D, JAR, IJ, MB, WLG‐J, FJB‐B and JJL analyzed the data and interpreted results of experiments; JAR drafted manuscript; RC, AM‐D and JJL edited and revised manuscript; AM‐D, JAR and JJL approved final version of manuscript.

### Peer review

The peer review history for this article is available at https://www.webofscience.com/api/gateway/wos/peer‐review/10.1002/1878‐0261.13629.

## Data Availability

All the data used are provided in this article.
